# Differential efficiency in exogenous DNA acquisition among closely related *Salmonella* strains: implications in bacterial speciation

**DOI:** 10.1186/1471-2180-14-157

**Published:** 2014-06-14

**Authors:** Hong-Xia Bao, Le Tang, Lu Yu, Xu-Yao Wang, Yang Li, Xia Deng, Yong-Guo Li, Ang Li, Da-Ling Zhu, Randal N Johnston, Gui-Rong Liu, Ye Feng, Shu-Lin Liu

**Affiliations:** 1 Genomics Research Center (one of The State-Province Key Laboratories of Biomedicine-Pharmaceutics of China), Harbin Medical University, 157 Baojian Road, Harbin 150081, China; 2 Department of Biopharmaceutical Sciences, Harbin Medical University, Harbin, China; 3 HMU-UCFM Centre for Infection and Genomics, Harbin Medical University, Harbin, China; 4 Department of Infectious Diseases of First Affiliated Hospital, Harbin Medical University, Harbin, China; 5 Department of Biostatistics, Harbin Medical University, Harbin, China; 6 College of Pharmacy of Daqing Campus, Harbin Medical University, Harbin, China; 7 Department of Biochemistry and Molecular Biology, University of Calgary, Calgary, Canada; 8 Department of Microbiology and Infectious Diseases, University of Calgary, Calgary, Canada; 9 Institute for Translational Medicine, Zhejiang University School of Medicine, Hangzhou, China

**Keywords:** Bacterial speciation, Homologous recombination, *Salmonella*, Transduction, Transformation

## Abstract

**Background:**

Acquisition of exogenous genetic material is a key event in bacterial speciation. It seems reasonable to assume that recombination of the incoming DNA into genome would be more efficient with higher levels of relatedness between the DNA donor and recipient. If so, bacterial speciation would be a smooth process, leading to a continuous spectrum of genomic divergence of bacteria, which, however, is not the case as shown by recent findings. The goal of this study was todetermine if DNA transfer efficiency is correlated with the levels of sequence identity.

**Results:**

To compare the relative efficiency of exogenous DNA acquisition among closely related bacteria, we carried out phage-mediated transduction and plasmid-mediated transformation in representative *Salmonella* strains with different levels of relatedness. We found that the efficiency was remarkably variable even among genetically almost identical bacteria. Although there was a general tendency that more closely related DNA donor-recipient pairs had higher transduction efficiency, transformation efficiency exhibited over a thousand times difference among the closely related *Salmonella* strains.

**Conclusion:**

DNA acquisition efficiency is greatly variable among bacteria that have as high as over 99% identical genetic background, suggesting that bacterial speciation involves highly complex processes affected not only by whether beneficial exogenous DNA may exist in the environment but also the “readiness” of the bacteria to accept it.

## Background

Speciation of bacteria, i.e., their divergence from the ancestor and evolution into new species, is facilitated by incorporation of laterally acquired genetic material into the genome, as demonstrated by comparative studies of model bacteria, such as *Salmonella* and other enteric bacteria [1-3]. Bacteria of the *Salmonella* genus have been frequently used as ideal research models of bacterial genomic divergence and evolution owing to several advantages that fit such studies, including the close genetic relatedness and, conversely, distinct pathogenic properties of these bacteria. In addition, the extraordinarily large number of known genetic and pathogenic types of *Salmonella* makes the comparative studies informative and feasible.

*Salmonella* diverged from *Escherichia coli* more than 100 million years ago [4-6]; to date, more than 2500 serologically defined types, called serotypes or serovars, have been documented [7,8]. Genomic comparisons reveal that different *Salmonella* serotypes have highly similar overall genome structures, which are also similar to that of *E. coli*[9-12], validating the long speculated high level genomic conservation during long evolutionary times. When more detailed comparisons are made systematically over the whole genome, specific differences can be found among even very closely related bacterial lineages [11,13-16]. Genomic differences can be classified essentially into two main categories: DNA sequence variations and distinct sets of insertions between the bacteria. It is well documented that monophyletic *Salmonella* serotypes (*versus* polyphyletic serotypes that contain genetically distinct lineages but are classified together due to their common serological features, such as *Salmonella paratyphi* B containing both single-host and broad-host infecting lineages that cause paratyphoid and gastroenteritis, respectively, in humans) may have “hallmark” insertions, such as the 134 kb SPI7 in most *S. typhi* isolates (*S. paratyphi* C and *S. dublin* have different versions of SPI7) [17-20]. Within a monophyletic *Salmonella* serotype, individual sub-lineages may also have their own unique insertions or combinations of them, as exemplified by the DT104 phage type of *S. typhimurium* for their possession of three insertions ST104, ST104B and ST64B, a combination not seen in other *S. typhimurium* sub-lineages [16,21]. All these facts suggest that, although bacteria have many chances to contact exogenous DNA in the environment and may even possibly have it moved into the cell via mechanisms such as bacteriophage-mediated transduction and plasmid-mediated transformation, the recipient may or may not have it incorporated into the genome regardless of whether or not the recipient might gain better fitness to the environment by having it. For example, although *in vitro* recombination efficiency depends linearly on the levels of the sequence similarity of the DNA strands, whether this linearity also exists in the exogenous DNA incorporation process inside a bacterial cell is unknown.

In this study, we assessed the efficiency of exogenous DNA acquisition by phage-mediated transduction and plasmid-mediated transformation in selected bacterial strains to establish whether DNA transfer efficiency might be correlated with the levels of sequence identity. We found that the efficiency was remarkably different among even very closely related bacteria. We conclude that DNA acquisition efficiency is greatly variable among bacteria that have as high as over 99% identical genetic background, which implies that bacterial speciation involves highly complex processes affected not only by whether beneficial exogenous DNA may exist in the environment but also the “readiness” of the bacteria to accept it.

## Results

### Transduction frequency and its correlation with sequence similarity: general tendency and strain-specific efficiency

**Figure 1:**
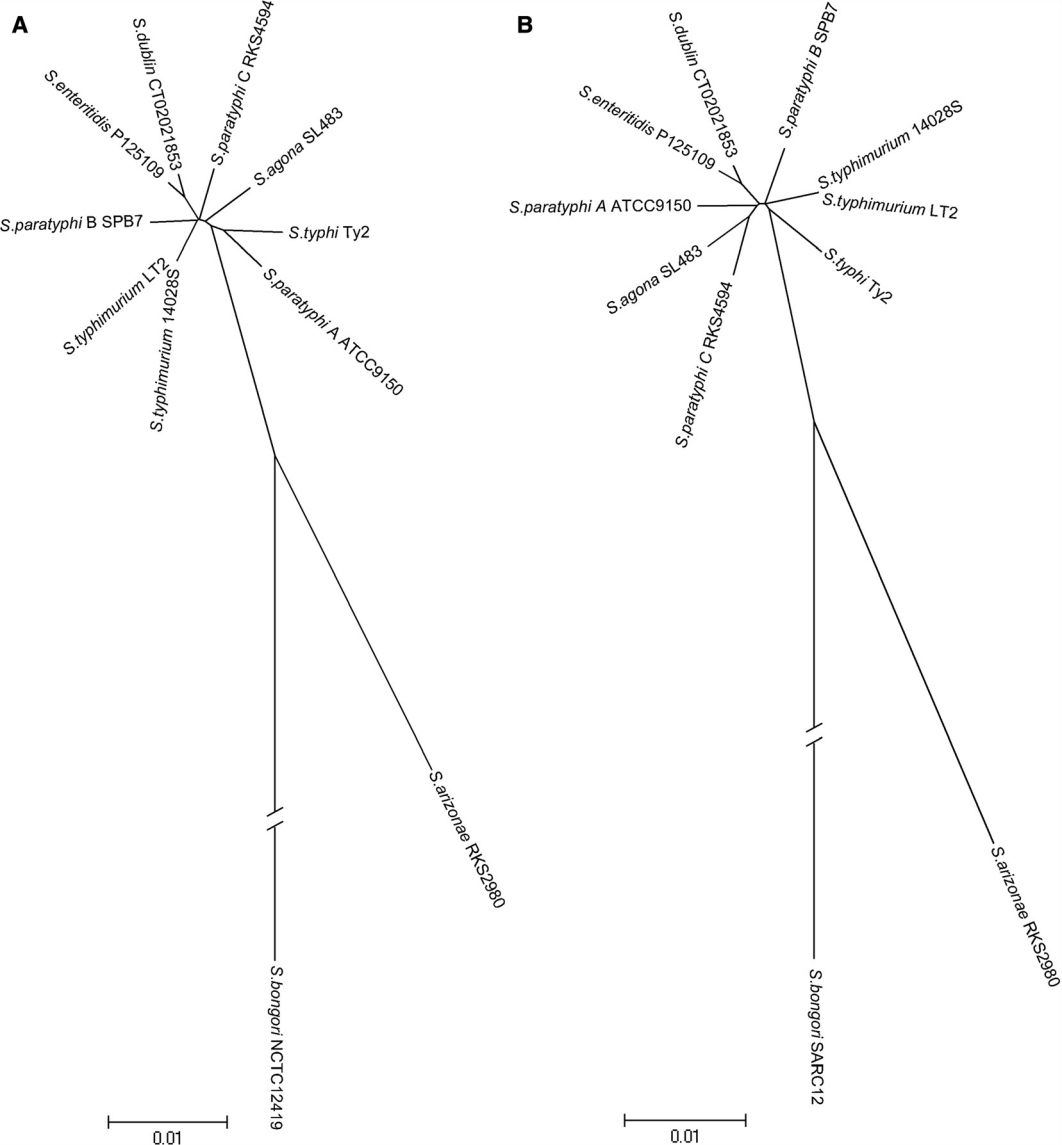
**Phylogenetic trees of *Salmonella *strains involved in transduction frequency comparison (see main text). A**, Phylogenetic tree based on core genes of the whole genome; **B**, phylogenetic tree based on the five genes.

We transferred five Tn*10*-inserted genes, including *flgL, treA, ompC, hisA* and *purG*, from *S. typhimurium* LT2 to the recipient strains (Table 1). To assess whether the five genes might be representative of the whole genome regarding the divergence among the *Salmonella* strains used in this study, we concatenated the sequences of the five genes and constructed a phylogenetic tree (Figure 1A) for comparison with the tree that was based on the core genes of the whole genome (Figure 1B). For the strains for which no whole genome sequences were available for tree construction, we used the published sequences of strains of the same serovar, such as *S. agona* strain SL483 instead of SARB1 (Table 1). The two trees demonstrated essentially the same genetic relatedness among the bacteria, justifying the use of these *Salmonella* strains, although the five genes had different levels of divergence among the *Salmonella* strains (Table 2).

**Table 1:** Bacterial strains used in this study*

**Strain**	**Accession number**	**Relationship with *S. typhimurium *** **LT2**	**Reference**
*S. typhimurium* LT2	AE006468.1	--	[38]
*S. typhimurium* 14028S	CP001363.1	Same serovar (1,4,[5],12:i:1,2)	
*S. typhi* Ty2	AE014613.1	Same subgroup (I)	[18]
*S. paratyphi* A ATCC9150	CP000026.1	Same subgroup (I)	
*S. paratyphi* B SPB7	CP000886.1	Same serogroup (04)	
*S. paratyphi* C RKS4594	CP000857.1	Same subgroup (I)	
*S. agona* SL483	CP001138.1	Same serogroup (04)	
*S. agona* SARB1	N/A	Same serogroup (04)	
*S. dublin*CT_02021853	CP001144.1	Same subgroup (I)	
*S. dublin* TYT3627	N/A	Same subgroup (I)	
*S. enteritidis* P125109	AM933172.1	Same subgroup (I)	
*S. enteritids* LK5	N/A	Same subgroup (I)	
*S. pullorum* RKS5078	CP003047.1	Same subgroup (I)	[23,24]
*S. gallinarum*287/91	AM933173.1	Same subgroup (I)	[25]
*S. arizonae* RKS2980	CP000880.1	Closely related subgroup (IIIa)	
*S. arizonae* SARC5	N/A	Closely related subgroup (IIIa)	
*S. bongori* NCTC 12419	FR877557.1	Distantly related subgroup (V)	
*S. bongori* SARC12	CP006692	Distantly related subgroup (V)	

*See more detailed information on these bacterial strains at http://www.ucalgary.ca/~kesander.

**Table 2:** S**equence divergence of the five genes between *S. typhimurium *** **LT2 and the recipient strains**

**Recipient strain**	***flgL***	***treA***	***hisA***	***ompC***	***purG***	**Average**	**SD**
*S. typhimurium* LT2	--	--	--	--	--	--	--
*S. typhimurium* 14028 s	0	0.0006	0	0	0	0.0001	0.0003
*S. paratyphi* B SPB7	0.0094	0.0093	0.0136	0.0088	0.0103	0.0103	0.0019
*S. agona* SARB1	0.0105	0.0146	0.0163	0.0202	0.0085	0.014	0.0047
*S. dublin* TYT3627	0.0084	0.0088	0.0081	0.0088	0.0082	0.0085	0.0003
*S. enteritidis* LK5	0.0126	0.0088	0.0122	0.0202	0.0082	0.0124	0.0048
*S. paratyphi* C RKS4594	0.0063	0.0128	0.0108	0.0202	0.0113	0.0123	0.0051
*S. paratyphi* A ATCC9150	0.0073	0.0123	0.0136	0.0053	0.0116	0.01	0.0035
*S. typhi* Ty2	0.0073	0.0169	0.0136	0.0053	0.0111	0.0108	0.0047
*S. arizonae* SARC5	0.0692	0.0736	0.0637	0.0325	0.0598	0.0598	0.0161
*S. bongori* SARC12	0.1143	0.0362	0.061	0.0554	0.0769	0.0688	0.0293

Recombination frequencies as reflected by the relative numbers of transductants on LB plates varied among thefive genes in a given recipient; however, the differences between strains were much greater (Table 3). As would be expected, *S. typhimurium* 14028S exhibited the highest frequency of recombination with the DNA from *S. typhimurium* LT2 among all recipient strainsand we did not get any transductants from *S. bongori *SARC12(the most distantly related species with *S. typhimurium* among all *Salmonella* lineages), althoughthis latter strain did express the O12 antigen after transformation by pPR1347 (See Methods). Notably, however, although 14028S has nearly identical genomic sequence to LT2, its recombination frequency was as low as merely one tenth that of LT2. A similar situation was seen with *S. enteritidis* LK5, whose sequence divergence is only 0.012 from *S. typhimurium* LT2 but its recombination frequency with LT2 was even lower than *S. arizonae* SARC5 with LT2, although *S. arizonae*is a serovar from subgroup IIIa, which is much more divergent than *S. enteritidis* from *S. typhimurium* (see Figure 1). Another notable observation was that we did not obtain any transductants from *S. typhi* Ty2 even though we tried numerous times, which however was consistent with our previous findings that we had to disrupt the *mutL* gene of *S. typhi* Ty2 in order to obtain transductants in the transduction experiments with the donor DNA from *S. typhimurium* LT2 when we were constructing a physical map for *S. typhi* Ty2 [18].

**Table 3:** **Relative transduction frequency of ***Salmonella ***strains for the five genes**

**Strain**	***flgL***	***treA***	***ompC***	***hisA***	***purG***	**mean ± sd**
*S. typhimurium* LT2	1	1	1	1	1	1
*S. typhimurium* 14028 s	1.17 × 10^-1^	0.60 × 10^-1^	0.92 × 10^-1^	0.79 × 10^-1^	0.73 × 10^-1^	(8.42 **±** 2.15) × 10^-2^
*S. paratyphi* B SPB7	0.42 × 10^-2^	0.24 × 10^-2^	1.03 × 10^-2^	1.36 × 10^-2^	1.68 × 10^-3^	(9.46 **±** 6.11) × 10^-3^
*S. agona* SARB1	0.66 × 10^-3^	0.11 × 10^-3^	0.61 × 10^-3^	1.45 × 10^-3^	0.15 × 10^-3^	(5.94 **±** 5.42) × 10^-4^
*S. dublin* TYT3627	4.30 × 10^-3^	1.92 × 10^-3^	3.64 × 10^-3^	3.50 × 10^-3^	3.29 × 10^-3^	(3.33 **±** 0.87) × 10^-3^
*S. enteritidis* LK5	2.20 × 10^-5^	0.88 × 10^-5^	0.00 × 10^-5^	1.80 × 10^-5^	1.05 × 10^-5^	(1.19 **±** 0.86) × 10^-5^
*S. paratyphi* C RKS4594	0.93 × 10^-3^	0.76 × 10^-3^	1.22 × 10^-3^	0.13 × 10^-3^	0.48 × 10^-3^	(7.02 **±** 4.19) × 10^-4^
*S. paratyphi* A ATCC9150	1.54 × 10^-4^	2.37 × 10^-4^	1.04 × 10^-4^	0.63 × 10^-4^	1.15 × 10^-4^	(1.35 **±** 0.66) × 10^-4^
*S. typhi* Ty2	<1.00 × 10^-6^	<1.00 × 10^-6^	<1.00 × 10^-6^	<1.00 × 10^-6^	<1.00 × 10^-5^	<1.00 × 10^-6^
*S. arizonae* SARC5	8.15 × 10^-4^	3.08 × 10^-4^	5.53 × 10^-4^	1.17 × 10^-4^	3.46 × 10^-4^	(4.28 **±** 2.66) × 10^-4^
*S. bongori* SARC12	<1.00 × 10^-6^	<1.00 × 10^-6^	<1.00 × 10^-6^	<1.00 × 10^-6^	<1.00 × 10^-6^	<1.00 × 10^-6^

Note: The relative transduction frequency listed in the table refers to relative transduction rate compared to LT2.

**Figure 2:**
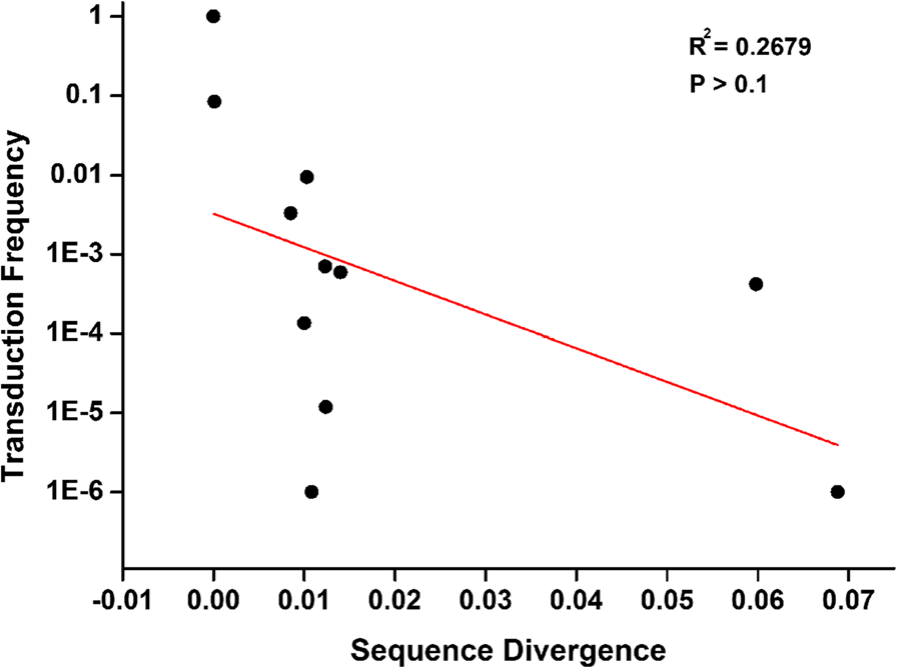
**Transduction frequencies against levels of relatedness among the *Salmonella *** **strains.**

As a whole in the experiments described above, although we saw a general tendency of higher transduction efficiency with closer genetic relationships between the donor and recipient bacterial strains, a linear model was not supported by our data (i.e., the relationship was not linear; Figure 2). Especially, the results out of *S. typhimurium* LT2 and 14028S seem to demonstrate that the transduction efficiency is rather strain-specific. We thus considered evaluating bacterial strain specificity by involving multiple strains of a *Salmonella* lineage and between very closely related lineages, i.e., *S. gallinarum* and *S. pullorum*.

### Transduction frequency between *S. gallinarum* and *S. pullorum*: variable efficiency among individual strains

**Figure 3:**
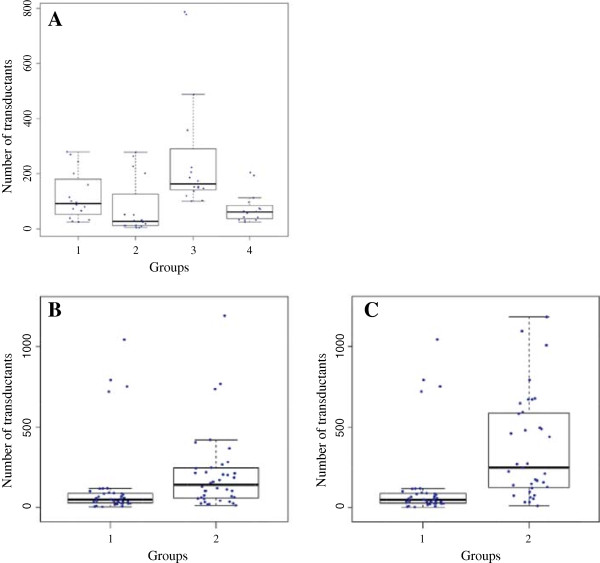
**Comparisons of recombination efficiency reflected by transduction frequencies, with the points representing the numbers of transductants. A**, Transduction of *ompD*159 between *S. gallinarum* and *S. pullorum* (see text for more details). Groups: 1, *S. pullorum* recipients and *S. pullorum* DNA; 2, *S. pullorum* recipients and *S. gallinarum* DNA; 3, *S. gallinarum* recipients and *S. gallinarum* DNA; 4, *S. gallinarum* recipients and *S. pullorum* DNA. Note that group 3 has the largest numbers of recipients (groups 2 vs 3, p = 0.007; groups 3 vs 4, p = 0.000), reflecting significantly higher recombination efficiency of *S. gallinarum* DNA into the *S. gallinarum* genome (group 3) than *S. gallinarum* DNA to the *S. pullorum* genome (group 2) or than *S. pullorum* DNA to the *S. gallinarum* genome (group 4). Group 1 had a similar tendency as group 3, although the differences were not statistically significant (groups 1 vs 2, p = 0.079; groups 1 vs 4, p = 0.071). **B**, Transduction with *S. pullorum*as the recipient and *S. pullorum* or *S. gallinarum*as the donor. Groups: 1, *S. pullorum* recipients with DNA from*S. gallinarum* 287/91; 2, *S. pullorum* recipients with DNA from *S. pullorum* RKS5078 (groups 1 vs 2, p = 0.009). **C**, Transduction with *S. pullorum* or *S. gallinarum*strains as the recipient and donor DNA from *S. gallinarum*. Groups: 1, *S. pullorum* recipients with DNA from *S. gallinarum* strain 287/91; 2, *S. gallinarum* recipients with DNA from *S. gallinarum* strain 287/91 (groups 1 vs 2, p = 0.000).

*S. gallinarum* and *S. pullorum* have a common antigenic formula, 1,9,12:-:-, the former causing typhoid and the latter causing pullorum disease in fowl. They were originally treated as separate species [22] but have since the mid 1980s been classified into the same serovar of the same subspecies (i.e., *S. enterica* subspecies *enterica* Serovar Gallinarum as separate biovars Gallinarum and Pullorum, respectively [8]). However, their distinct biological properties, i.e., causing entirely different diseases, unambiguously distinguish them as different organisms. Recently our work reveals that the two pathogens have accumulated distinct sets of mutations, including different pseudogenes [15,23], which further demonstrated their genetic divergence. We moved the Tn*10-*inserted *ompD* gene first from *S. typhimurium* LT2 to four wild type strains each of *S. pullorum* (including strain RKS5078 [23,24]) and *S. gallinarum* (including strain 287/91 [25],) and then from one of the eight strains to the other seven strains; this process was repeated for each of the eight strains. We observed a tendency that transduction frequency was higher in a donor-recipient pair of strains within *S. pullorum* or within *S. gallinarum* than between *S. pullorum* and *S. gallinarum* strains, although not all of these differences were statistically significant (Figure 3A). To validate this observation and rule out the possibility that a particular genomic DNA segment (e.g., *ompD* in Figure 3A) or some bacterial strains might have biases, we used Tn*10*-inserted *leu*, *bio*, *oxrA* and *cysA* as donor DNA, with *ompD* also included as donor DNA for a comparison, in the second set of transduction experiments with larger numbers of *S. pullorum* and *S. gallinarum* strains. Again, the transduction frequency was lower between *S. pullorum* and *S. gallinarum* than within *S. gallinarum* or *S. pullorum*, but, similar to the above experiments, not all of the differences were statistically significant (Figure 3B & C).

### Transformation efficiency among individual *Salmonella* strains

As transduction frequency reflects a combined result of DNA taking up capability and recombination efficiency, we wondered whether the non-linearity of transduction frequency with the level of relatedness of the bacteria might have mainly resulted from differential capability of the bacteria to take up DNA from the environment into the cell. To look into this, we transformed representative strains with the plasmid pQE30, which does not have homologous sequences with the genomes of the bacterial strains used in the study, and compared the transformation efficiency among them. Intriguingly, the transformation rates differed more than one thousand times among the tested bacteria, e.g., as low as ca. 8.3 × 10^-7^ in* S. enteritidis* LK5 andas high as ca.1.3 × 10^-3^in*S. arizonae* SARC5 (Table 4).

**Table 4:** Frequencies of transformation among the recipient strains for the same donor DNA

**Strain**	**Transformation frequency**
*S. typhimurium* LT2	(4.30 **±** 1.15) × 10^-4^
*S. typhimurium* 14028S	(5.08 **±** 0.72) × 10^-6^
*S. paratyphi* B SPB7	(4.10 **±** 1.40) × 10^-4^
*S. agona* SARB1	(2.25 **±** 0.90) × 10^-6^
*S. dublin* TYT3627	(2.46 **±** 0.91) × 10^-4^
*S. enteritids* LK5	(8.33 **±** 1.44) × 10^-7^
*S. paratyphi* C RKS4594	(1.44 **±** 0.01) × 10^-4^
*S.paratyphi* A ATCC9150	(2.93 **±** 1.05) × 10^-4^
*S. typhi* Ty2	(8.15 **±** 1.43) × 10^-4^
*S. arizonae* SARC5	(1.32 **±** 0.51) × 10^-3^
*S. bongori* SARC12	(1.58 **±** 0.45) × 10^-3^

## Discussion

Two classes of genomic changes are associated with the phylogenetic divergence and evolution of bacteria – acquisition of laterally transferred DNA and nucleotide substitution, with the former being the primary driver of bacterial speciation, i.e., the process and consequence of bacterial development into a nascent biological species.

The biological species is defined on the basis of sexual reproduction potential of the organisms [26,27], i.e., each species is separated from others through reproductive barriers. This definition of species works well for many plants and animals but not so much for bacteria owing to the mostly non-sexual reproduction modes of bacteria. As bacteria acquire much of the genetic novelty through homologous recombination of laterally transferred DNA, it seems reasonable to presume that recombination efficiency among bacteria should be falling gradually with increasing sequence divergence. Several lines of evidence exist to support this presumption in *Escherichia, Bacillus* and *Streptococcus*[28-30]. If so, bacterial speciation would be a gradual process, leading to continuous divergence of the bacterial genomes without genetic boundaries to delineate bacteria into discrete clusters, i.e., bacterial species would have to be arbitrary. However, our recent work has demonstrated the existence of clear-cut genetic boundaries among bacteria as closely related as the *Salmonella* lineages [31], indicating that a fundamental question still remains regarding molecular mechanisms involved in bacterial speciation, especially the DNA acquisition capability of the bacteria.

Based on our hypothesis of genetic boundaries and the experimental evidence [31], we propose that biological species should be non-continuous and non-overlapping units of organisms with sufficient similarities among members of the same species and radically distinguishing features from organisms of other species. Bacteria, though as essentially non-sexual organisms, should also be genetically isolated from one another as species. To date, thousands of bacterial strains have been sequenced, with a full picture of the genomic divergence among them awaiting systematic analyses. We believe that genomic divergence among bacteria is not continuous and clear-cut boundaries ought to exist to demarcate bacteria into discrete species. In our Adopt-Adapt Model of bacterial speciation [17,32,33], we dissect the process into two overall stages: Adopt, in which bacteria “adopt” exogenous DNA from the environment to gain beneficial genetic traits, and Adapt, in which bacteria make adjustments to “adapt” to the genetic and biological changes brought about by the novel genetic material, including genomic rearrangements (if the adopted DNA segment was large such as larger than 100 kb [34]) and genetic separation from the ancestral strain as reflected by the formation of the genetic boundaries [31]. According to this model, exogenous DNA acquisition is the first step; the present study demonstrates that the stochastic lateral DNA acquisition events may not be destined to take place even though the “right” recipient might meet the “right” donor at the “right” time (e.g., when the recipient is in bad need of certain trait that could be donated by a potential donor nearby in the environment). Rather, this event might be under strict control, although the molecular mechanisms are largely unknown. In this study, we assessed correlations between genomic sequence divergence and transduction or transformation frequency among closely related bacteria, anticipating either (i) a linear model with falling transduction or transformation frequency in correlation with increasing genomic divergence; or (ii) a non-linear model; we observed the latter.

To interpret these observations, we may divide the exogenous DNA acquisition process into two steps. In the first step, exogenous DNA enters the cell and escapes degradation by restriction enzymes or other defense systems. The next step includes hetero duplex molecule formation, replication and segregation to daughter cells. This second step is controlled negatively by mismatch repair (MMR) proteins, such as *MutS* and *MutL*, and positively by the induction of SOS system. As MMR and SOS genes are highly conserved in bacteria, they are supposed to function equally in all tested *Salmonella* strains. Consequently, the second step here should mainly be determined by the sequence divergence between the donor and recipient. It is therefore reasonable to presume that the non-linear correlation between recombination efficiency and genomic divergence should be due to differences in the first step, i.e., entry of exogenous DNA into the bacterial cell. As transduction and transformation use different mechanisms to bring DNA into the cell across the cell wall and membrane, the plasmid transformation may not effectively simulate the first step of P22-mediated homologous recombination. Nevertheless, when we divided the transduction frequency by the transformation frequency in a given strain, the “normalized” results fitted the linear model slightly better (calculation not shown). Taken together, our results demonstrate that the linear model may hold true only when recombination efficiency is determined exclusively by sequence divergence, although there seems no such a case in the real world. As this study focused on only DNA transfer events, systematic elucidation of the bacterial speciation mechanisms requires investigation of also other strain differences such as level of gene expression for competency/DNA recombination, which is under study in our laboratory. Since transformation and transduction are the two main mechanisms for DNA uptake by bacteria and even very closely related bacteria may exhibit distinct performance in recombination as demonstrated in this study, it is possible that subpopulations or even individual cells of a bacterial species might have begun to form genetic barriers against exogenous DNA to stabilize the bio-system within the cell or a subpopulation or to continue the speciation process to become a nascent species.

## Conclusion

DNA acquisition efficiency is variable among closely related bacteria, suggesting that bacterial speciation involves highly complex processes affected not only by whether beneficial exogenous DNA may exist in the environment but also the “readiness” of the bacteria to accept it.

## Methods

### Bacterial and phage strains

The *Salmonella* strains used in this study are listed in Table 1; more detailed information on these bacterial strains can be found at the *Salmonella* Genetic Stock Center (SGSC; http://www.ucalgary.ca/~kesander/). The bacterial strains were cultured at 37°C and the bacteriophage P22 strain (HT105/1 int-201) used in the transduction experiments was routinely grown on *S. typhimurium* LT2.

### Phage P22-mediated transduction

P22-mediated transduction was used to mediate homologous recombination between donor and recipient strains as previously described [35,36]. The primary donor strains were derivatives of *S. typhimurium* LT2 [37,38]. Five such derivative strains were used, having Tn*10* (with tetracycline resistance) inserted within gene *flgL, treA, ompC, hisA* or *purG*. After P22 infection of the secondary donor strains (*S. gallinarum* or *S. pullorum* strains used as donors of the Tn*10*-inserted genes to the *S. gallinarum* or *S. pullorum* recipient strains; see Results), the phage lysate was used to infect recipient strains. Construction of the secondary donor strains from the Tn*10*-inserted LT2 derivatives and preparation of phage lysate were done as described [36].

As P22 can only infect the bacteria that express the O12 antigen, we first transformed the O12^-^recipient strains by a cosmid, pPR1347, that carries the O12 antigen gene [39]; details of the experimental procedure can be found in literature [36]. Briefly, the *E. coli* strain that carries pPR1347 was cultured in LB broth overnight. The cosmid was extracted by alkaline lysis method and was transferred into *S. paratyphi* C RKS5478, *S. arizonae* SARC5 and *S. bongori* SARC12; all of the three bacterial strains expressed O12 after the transformation by pPR1347.

The transduction frequency was determined as follows. The recipient strains were cultured in LB broth till OD600 reaching 2.0. Then the bacteria were mixed with P22 phage lysate at an MOI (multiplicity of infection) of 0.01, plated onto LB medium containing tetracycline (15 μg/ml) and incubated at 37°C for 18 h. Because all recipient strains were sensitive to tetracycline, only clones that have gone through successful homologous recombination would be able to grow on the tetracycline-containing plate. The relative transduction frequency of each recipient strain was calculated by dividing the number of its transductants by the number of LT2 transductants transduced by the same lot and amount of the phage lysate. Each experiment was repeated five times.

### Transformation

The plasmid pQE30, which carries ampicillin resistance, was used for transformation. The recipient bacteria were cultured in LB broth until OD600 reached 0.4. Competence was induced by thawing the culture on ice for 1 min. A volume of 80 μl competent cells were mixed with 2 μl plasmid pQE30 and electroporated at 25 μF capacitance, 2.5 kv and 200 ohm resistance, followed immediately by the addition of 320 μl fresh LB medium. The bacteria were incubated on 37°C with agitation of 85 rpm for 1 hour. Then the bacteria were spread onto LB agar plates containing ampicillin (100 μg/ml) and incubated at 37°C overnight. As all recipient strains used were sensitive to ampicillin, only successful transformants could grow on ampicillin plates. The transformation frequency was calculated by dividing the number of transformants by the number of competent cells of the same volume grown on pure LB plate. Each experiment was repeated five times.

### Calculation of sequence divergence

The genome sequences for comparison were downloaded from Genbank database, with the accession numbers being listed in Table 1. The genome sequence of *S. agona* SARB1, *S. dublin* TYT3627, *S. enteritidis* LK5 are not available in public database yet, so we use the published strains of the same serovar instead (see Table 1). Because *S. bongori* SARC12 doesn’t have any published relative of the same serovar, we sequenced its genome by using SOLiD™ ^3.0^ sequencer. Briefly, we sheared the bacterial genomic DNA into fragments of 2 ~ 4 kb in size and sequenced both ends of the fragments following SOLiD™ ^3.0^ 2 × 50 bp mate-pair sequencing protocol. The raw reads were assembled into draft genome by using the software SOLiD™ System *de novo* Accessory Tools 2.0 (http://solidsoftwaretools.com/gf/project/denovo/). The gaps between the obtained contigs were closed by PCR.

We calculated the sequence divergence between the compared strains by two approaches. The first approach was based on five genes only. The nucleotide sequences of *flgL, treA, ompC, hisA* and *purG* were extracted from the complete genomes, aligned by CLUSTALW, and then concatenated together. The alignment was input into MEGA5 for constructing the phylogenetic tree. The Jukes–Cantor substitution model was adopted and the neighbor-joining algorithm was implemented. The second approach was based on all conserved orthologous genes. We chose LT2 as the reference genome and searched its protein-coding sequences against other strains by BLAST. The threshold of the BLAST search was as follows: the e-value must be smaller than 1 × 10^-10^, the identity of the amino acid must be larger than 80% and the alignment length must be longer than 80% of the gene length. The conserved genes among all strains were then aligned by CLUSTALW and were concatenated together. Finally the alignment was input into MEGA5 and the construction of the phylogenetic tree was built with the same parameter to the first approach.

## Availability of supporting data

The genome sequence of *S. bongori* SARC12 is available the Genbank database under the accession number CP006692. The phylogenetic trees supporting the results of this article are available in the TreeBASE repository, http://purl.org/phylo/treebase/phylows/study/TB2:S15939?x-access-code=26f7bb0963bf24cff2e944fe3065c5ec&format=html.

## Competing interests

The authors declare that they have no competing interests.

## Authors’ contributions

HXB and LT carried out the experimental work and genomic sequence analysis. LY, XYW, YL and XD participated in the experimental work. AL conducted statistical analysis. RNJ, YGL, DLZ, YF and GRL participated in data analysis. YF participated in the designing of the study. HXB, LT and YF contributed to drafting the manuscript. SLL conceived of and designed the study and wrote the manuscript. All authors read and approved the final manuscript.
